# Stigma and Discrimination (SAD) at the Time of the SARS-CoV-2 Pandemic

**DOI:** 10.3390/ijerph17176341

**Published:** 2020-08-31

**Authors:** Antonio Baldassarre, Gabriele Giorgi, Federico Alessio, Lucrezia Ginevra Lulli, Giulio Arcangeli, Nicola Mucci

**Affiliations:** 1 Doctoral School in Clinical Sciences, University of Florence, 50134 Florence, Italy; 2 Occupational Medicine Unit, Careggi University Hospital, 50134 Florence, Italy; 3 Department of Human Sciences, European University of Rome, 00136 Rome, Italy; prof.gabriele.giorgi@gmail.com (G.G.); federico.alessio94@gmail.com (F.A.); 4 Department of Experimental and Clinical Medicine, University of Florence, 50134 Florence, Italy; lucreziaginevra.lulli@unifi.it (L.G.L.); giulio.arcangeli@unifi.it (G.A.); nicola.mucci@unifi.it (N.M.)

**Keywords:** SARS-CoV-2, COVID-19, epidemic outbreak, pandemic outbreak, infectious disease, Stigma, Discrimination, avoiding behaviors

## Abstract

Infectious disease control is a crucial public health issue. Although it is important to urgently perform public health measures in order to reduce the risk of spread, it could end up stigmatizing entire groups of people rather than offering control measures based on sound scientific principles. This “us” versus “them” dynamic is common in stigmatization, in general, and indicates a way in which disease stigma can be viewed as a proxy for other types of fears, especially xenophobia and general fear of outsiders. The pandemic risk associated with SARS-CoV-2 infection led us to consider, among other related issues, how stigma and discrimination remain serious barriers to care for people suspected of being infected, even more if they are assisting professions, such as health workers, employed in emergency response. The purpose of this review is to evaluate and promote the importance of psychological aspects of the stigma and social discrimination (SAD) in pandemic realities and, more specifically, nowadays, in the context of SARS-CoV-2/COVID-19. Just as it happened with HIV, HCV, tuberculosis, and Zika, stigma and discrimination undermine the social fabric compromising the ethics and principles of civilization to which each individual in entitled. Recognizing disease stigma history can give us insight into how, exactly, stigmatizing attitudes are formed, and how they are disbanded. Instead of simply blaming the ignorance of people espousing stigmatizing attitudes about certain diseases, we should try to understand precisely how these attitudes are formed so that we can intervene in their dissemination. We should also look at history to see what sorts of interventions against stigma may have worked in the past. Ongoing research into stigma should evaluate what has worked in the past, as above-mentioned, providing us with some clues as to what might work in the current pandemic emergency, to reduce devastating discrimination that keeps people from getting the care they need. We propose a systematic and historical review, in order to create a scientific and solid base for the following SAD analysis. The aim is to propose a coping strategy to face stigma and discrimination (SAD) related to SARS-CoV-2/COVID-19 pandemic outbreak, borrowing coping strategy tools and solutions from other common contagious diseases. Furthermore, our study observes how knowledge, education level, and socioeconomic status (SES) can influence perception of SARS-CoV-2/ COVID-19 risk in a digital world, based on previous research, best practices, and evidence-based research.

## 1. Epidemics Outbreak and Society, an Historical View

According to work by Rosenborg [1] (1989), inspired by the Albert Camus novel published in 1947, epidemic outbreaks can be divided into three social phases. First, the society, seeking to reassure itself and to safeguard its economic interests, tries to reject the existence of epidemics. This phase continues if the community is reluctantly forced to face illness and mortality acceleration. The first act brings acknowledgement, but not acceptance, and this process leads to the second step. In the following phase, people demand and offer explanations, both mechanistic and moral. These aggregate explanations form the “public” responses, which can hide pitfalls and problems and make the third phase as dramatic and disruptive as the disease itself. One of the most important aspects of this process is the desire to assign personal and social responsibility (Rosenborg, 1989, according to Terror Management Theory by Greenberg et al., 1997 [2]; the prejudice theory of Allport, 1958, [3]). The research, of some kind of guilt and blame, exploits existing social divisions of religion, race, ethnicity, class, or gender identity. According to the David Jones [4] historical review (2020), two familiar aspects echo in the history of pandemics. First, stigmatization and discrimination follow closely on the heels of every pathogen. For example, anti-Chinese hostility has been a recurrent problem, i.e., with the plague in San Francisco in 1900, SARS in 2003, or COVID-19 today. Secondly, epidemics too often claim the lives of healthcare providers (Jones [4], 2020).

## 2. Introduction to Stigma and Discrimination

Even if the research about stigma was created earlier, the first full description of the stigma was given by Goffman’s work [5], *Stigma: Notes on the Management of a Spoiled Identity*, in 1963. Stigma is a social label that bans subjects from the full acceptance of the society in which they live (Goffman [5], 1963, p. 3), and it is defined as “an attribute that links a person to an undesirable stereotype, leading other people to reduce the bearer from a whole and usual person to a tainted, discounted one (p. 11)”.

Crocker, Major, and Steele (1998) [6] further highlighted the contextual nature of stigma: several attributes can be condemned from a part of the society but accepted from other groups or minorities. This theory partly follows the study of S. Moscovici [7] in the classic work *L’âge des foules*, dated 1981. In fact, sometimes, when researchers refer to stigma as a stressor, they are referring to the anticipation of negative treatment by members of dominant groups, Meyer, 2003 [8].

Another key element of stigma is power: “Stigmatization is entirely contingent on access to social, economic, and political power that allows the identification of differentness, the construction of stereotypes, the separation of labeled persons into distinct categories, and the full execution of disapproval, rejection, exclusion, and discrimination” (Link and Phelan, 2001, p. 367 [9]). Stigma is something that is socially conferred—that both signals the recognition of difference and devalues the person [10].

The functions of stigma and prejudice in society have varied throughout history. While the ancient causes of stigma and prejudice originated from exploitation and dominance, nowadays stigmatization is socially used to fortify undisputed regulations for the majority, enforce accepted behaviors, and permit disease avoidance [11].

Stigma has some strength similarities to “prejudice”, according to work by Allport (1958) [3]. The differences between the research traditions of stigma and prejudice have more to do with different subjects of interest rather than any real conceptual difference [12]. In fact, stigma research has traditionally emphasized the study of people with unusual conditions and visible differences (for example, facial disfigurement, HIV/AIDS, short stature, and mental illness) [12]. Stigmatization usually leads to discrimination [12,13]; consequently, discrimination brings unequal behaviors from the collectivity and the society, convoying high levels of individual stress and significant health disparities [14]. In their research, Dovidio et al. [15] (2010) focused on three stigmatized groups (blacks, sexual minorities, and people suffering obesity), underlining that stigmatization is related to lower and poorer health levels. Kowalczuk et al. (2018) [16] also highlighted the existence of a horizontal discrimination in such occupational sectors, which typically offer lower rates of wages, and where women are frequently over-represented.

Nowadays, stigma and discrimination (SAD) is a current topic in the workplace, where it may cause negative repercussions for individuals (such as mental health problems), as well as for companies [17]. It follows the need to study this theme, also, from the point of view of occupational medicine and hygiene, to develop preventive programs aimed at improving global workplace health [18].

Stigma and discrimination have been, and currently are, major issues during the SARS-CoV-2 pandemic. Specific tools are needed to investigate their impact on sick people, as well as healthcare workers dealing with COVID patients. So far, no studies have been conducted on this specific psychological impact related to SARS-CoV-2. The purpose of this review is to offer a psychological driven perspective on the SARS-CoV-2 pandemic in relation to stigma and discrimination, based on consolidated scientific evidence gained through the study of other infectious diseases. The results will provide accurate insight on the various aspects of stigma and discrimination related to infectious diseases to be applied to the current pandemic scenario.

## 3. Material and Methods

The aim of this study is to evaluate and promote the importance of psychological and social aspects, such as education levels, personality, and socioeconomic status, in the context of SARS-CoV-2, in order to face stigma and discrimination (SAD) risk.

Below, the Systematic Review’s Problem, Intervention, Comparison, Outcome (PICO) research strategy (Table 1).

Therefore, we have conducted a review of the scientific literature carried out by both academic and professional backgrounds, and used the Preferred Reporting Items for Systematic Reviews and Meta-Analyses (PRISMA) 2009 Checklist Model.

### 3.1. Literature Research

The search strategy used a combination of controlled vocabulary and free text terms based on the following keywords:

Stigma or stigmatization, discrimination or discriminatory behavior or prejudice and SARS-CoV-2 or COVID-19.

All research fields were considered. Additionally, we practiced a manual search on reference lists of the selected articles and reviews to carry out a wider analysis.

Two independent reviewers read the titles and abstracts of the reports identified by the search strategy. They selected the relevant reports according to the inclusion and exclusion criteria reported below. Doubtful reports were discussed with a third separate researcher. Subsequently, the researchers independently screened the corresponding full text to decide on final eligibility. Duplicate studies and articles without full texts were eliminated.

### 3.2. Inclusion and Exclusion Criteria

**Figure 1:**
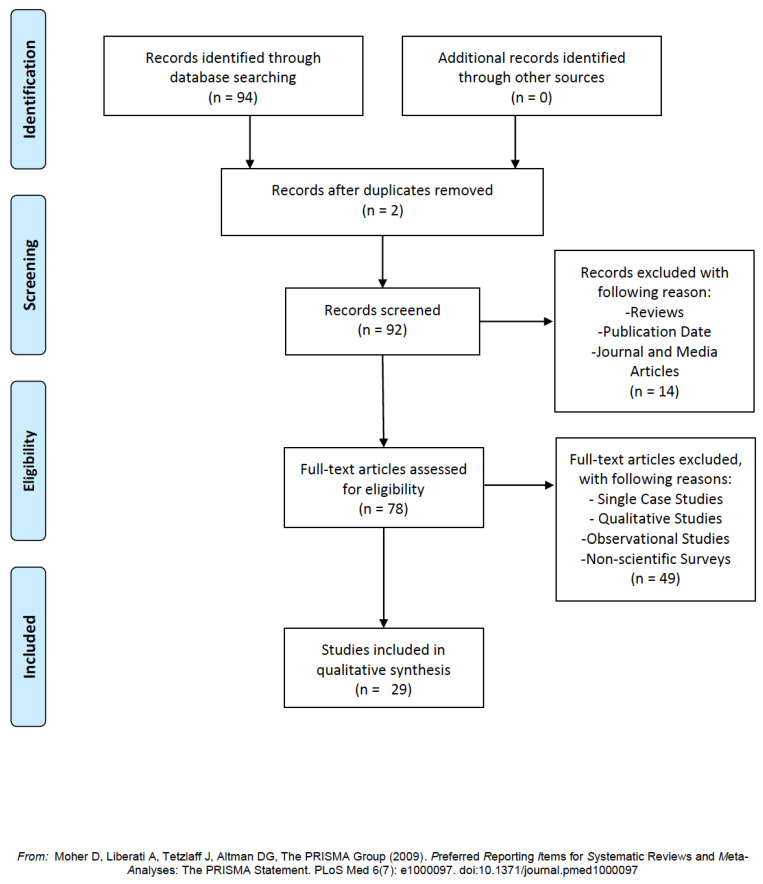
Preferred Reporting Items for Systematic Reviews and Meta-Analyses (PRISMA) flow-chart.

In our inclusion criteria, all research from the last twenty years (2000–2020) was taken into consideration. All unscientific or excessively qualitative research has been excluded, and precise inclusion criteria established. We excluded other literature reviews, qualitative or one-subject clinical research, journals, or other informational non-scientific papers. We included only medical, psychological, and socioscope research with significant samples, results, and evidence-based analysis (Table 2). From PubMed and the Google Scholar scientific database, 94 articles were analyzed and 29 selected, responding to the previously mentioned criteria set (Figure 1).

## 4. The Roles of Knowledge and Awareness in Epidemic Related Stigma

In the early days, the response to stigma had an innate and adaptive function related to the survival of the species; its role was to protect human beings from the spread of contagious and potentially fatal diseases [11]. In fact, a study made by Kouznetsova and colleagues (2011) [19] highlighted the evolutionistic role of the stigma: avoid the disease spread, through the social estrangement of those who carry infectious diseases, or who appear to be infectious with visible conditions [10,12,19].

Therefore, just like the fear response [20], or automatic/heuristic behaviors [21], and other systemic cognitive and emotional patterns, the instinctual response to stigma can bring to bias. These biases are naturally prerogative of the human being, but they can also be fertile ground for maladaptive and discriminatory behaviors. Lack of knowledge, or excessive instinct, are the main drivers of bias and discriminatory behaviors [21].

A socioscope survey conducted by Samuel G. and colleagues, in 2018 [22], studied the importance of knowledge in order to face epidemics and pandemics, particularly Zika virus outbreak disasters. Zika virus (ZIKV) is a single-stranded RNA virus of the Flaviviridae family, transmitted primarily through the bite of an infected Aedes species mosquito. The first large outbreak of the Zika virus occurred on the island of Yap in 2007, when an estimated 73% of the population contracted the virus. Another epidemic flood appeared in Brazil in 2015. However, Zika virus had already been discovered and isolated in 1947 [23]. In this socioscope survey, there were 224 participants with a mean age of 33 (SD ± 11.6), 77% female and 24% of them pregnant. The majority of the sample (98%; 213/217) were unable to identify all of the symptoms associated with acute Zika virus infection and all modes of transmission (97%; 213/219). The most concerning aspect of Zika virus for 46% of the sample (91/200) was the risk of disabilities to babies, and risk of sexual transmission (25%; 49/200). Most participants (80%; 165/207) believe that Zika virus was an important issue in their community, a quarter (25%; 51/201) of all participants and 36% (16/45) of pregnant participants thought that a person who had Zika virus, and their family, would face stigmatization. Similarly, 30.2% (63/197) of all, and 46% (20/44) of pregnant participants indicated that if a woman had a baby with microcephaly or another disability, she would be stigmatized because of the child. The results suggest that while the majority of participants heard of Zika prior to this survey, there are gaps in knowledge and information regarding multiple aspects of the Zika virus, including transmission risks, prevention methods, and symptoms. Following these arguments, the population can be harmed by the disease itself, but also by stigmatization and discrimination, enforced by prejudice and low levels of knowledge. In fact, other research noticed a powerful relation between knowledge and stigmatization (i.e., Milin et al., 2016 [24], in their studies on mental illness and stigma, and Herek et al., 2005 [25] in “HIV-Related Stigma and Knowledge in the United States”). Raising awareness of the risks coming from chronic, epidemic, or mental illness, and from new infectious diseases, will help promote social recommended treatment regimens and lower levels of discrimination (i.e., [26,27]).

Stigma does not only produce discriminatory behavior and estrangement between family and friends [28] (therefore, crumbling the foundations of the social fabric), but it also spreads individual issues, such as the perception of a negative self-image [27]. Findings show that stigma affects large groups of population and takes various forms: stigmatized people are shunned, insulted, marginalized, and rejected in the domains of work, interpersonal relationships, use of services and schooling [15,29].

## 5. Results

The articles that met the PICO criteria adopted for this systematic review are summarized in Table 3.

## 6. Discussion

The stigma related to some of the most common infectious pathologies (in the past and in the current century), such as human immunodeficiency virus (HIV), hepatitis C virus (HCV), tuberculosis, and Zika, is present at all levels, and acts as a critical barrier for effectively addressing it. This may also influence the treatment uptake, and under- or non-participation in treatment available [30], in particular in those countries where access to care is still affected by the effects of the global economic crisis of the past decade [53].

Even medical conditions that are non-contagious, but that appear contagious, seem to result in an attempt to avoid the sufferer by society. Error management theory (EMT) suggests that such false alarms occur because the cost of infection poses a greater threat to one’s fitness than avoidance [19]. For this reason, this idea shows how humans have evolved toward a general tendency to avoid individuals with disease signs, especially if displayed upon the face. One consequence is that where a facially displayed disease sign persists, even if known to be benign, its bearer will experience chronic avoidance [19].

In view of this, the study by Jain et al. aimed to assess the stigma of otherwise healthy individuals of the community toward HIV infection/Acquired Immuno-Deficiency Syndrome (AIDS) [30].

The study was conducted on 100 healthy participants, divided into two groups based on their levels of education (<12 years of formal education and >12 years of formal education). Stigma related to HIV/AIDS was compared among these two groups, and there was no significant difference in the level of stigma in these distinctly different educational groups. The results showed that there is more perceived stigma as compared to enacted stigma. Nearly 46% of the individuals feel that HIV-infected persons should be blamed for their illness and 41% individuals feel that they will feel ashamed if they have HIV. It was also seen that older adults (between 46 and 55 years) have more stigma as compared to younger adults (between 16 and 25 years). The most educated individuals still have stigma to a certain extent. Most of the individuals would like to tell their partners if they were diagnosed with HIV. This study shows how today, stigma is still present in society, to a certain extent, among educated and urban individuals. The level of stigma may not be significantly different in people with educational differences [30].

In this regard, Drewes et al. found evidence for the significant role of perceived contagiousness in HIV-related stigma, and were able to experimentally demonstrate the potential of antiretroviral therapy (ART) to reduce HIV-related stigmatization by lowering viral load and contagiousness, when these changes are accompanied by decreased perception of contagiousness [42].

Perceived contagiousness is a major dimension underlying HIV-related stigmatization. To test the assumption that reductions in contagiousness can lead to a decrease in stigmatizing reactions, Drewes et al. [42] conducted an experimental online study. A sample of 752 participants (50.9% female) read a short vignette depicting an HIV-positive individual with either a high or a low viral load, and were either given, or not given, information about the association between viral load and contagiousness. Subsequently, participants were asked to rate their willingness to stigmatize this individual by responding to two measures of social and physical distancing. Planned contrast analyses confirmed that physical distancing in the informed group was lower in the low viral load condition compared to the high viral load condition [42].

In his study, with a sample of 13 HIV-positive patients diagnosed at most six months before being interviewed, Fatemeh G.H. (2019) [32] and colleagues found that stigmatizing attitudes and discriminatory behaviors of other people, such as relatives and people in society, had a negative impact on the participants’ mental status, as well as on their perceptions and understanding about the medical diagnosis. Thus, the focus of care for people with HIV/AIDS should shift from therapeutic issues to socio-cultural ones. Even epilepsy studies (i.e., [31]) highlighted the importance of a social approach dedicated to raising and boosting awareness and social knowledge about the disease. Furthermore, Dar’s study (2018) [35] also underlined the importance of reducing psychological distress in patients with tuberculosis (PTB), highlighting how quality of life is also significantly reduced in patients with PTB, and how it improves rapidly and significantly after directly observed treatment, short-course (DOTS)-based intensive phase of treatment.

## 7. Conclusions

In conclusion, the studies analyzed in this paper suggest that stigmatization has important implications for both mental and medical health of the people affected by infectious diseases. Improving knowledge and awareness about these infectious diseases in society could lead to a significant improvement in a patient’s well-being.

Finally, our review highlighted how the scientific community has paid attention to such psychological aspects linked to certain morbid conditions, such as communicable infectious diseases, only in the last decade, in spite of the immediately preceding period. A multidisciplinary approach, which also contemplates the management of these psychological aspects, is to be considered mandatory by health policy makers.

Best evidence suggests all-round management, setup by medical, psychological, and social cooperation, led by health policymakers and planners. This seems to be the most appropriate approach to face the stigma and discrimination (SAD) related to the current SARS-CoV-2 pandemic outbreak.

## Figures and Tables

**Table 1:** Problem, Intervention, Comparison, Outcome (PICO) research strategy.

P	Problem	How stigmatization and discriminatory behaviors affect the population during and following a pandemic outbreak? What can we learn from the past? What will be the most suitable tools and practices in dealing with stigma?
I	Intervention	What is being done for stigmatized individuals and what is being done for dealing with discriminatory behavior? How can knowledge and awareness of the disease influence the behavior?
C	Comparison	Difference between different tools, practices, and methodologies.
O	Outcome	Find the best practice to enforce psychological and social health after a pandemic outbreak.

**Table 2:** Inclusion and exclusion criteria.

Inclusion Criteria	Exclusion Criteria
Scientific research from PubMed, Scholar, and Scopus databases. Medical, psychological research, and evidence-based socioscope surveys. Scientific quantitative research with significant samples (more than 10 participants). Research edited from 2000 to 2020.	Non-scientific surveys or only qualitative research. Journal and media articles or data, humanitarian organizations articles, observational studies, and reviews. Single case/one-participant and qualitative studies. Research published before 2000.

**Table 3:** Analysis of the literature.

Title	1st Author	Year	Discussion
A questionnaire survey of stigma related to human immunodeficiency virus infection/acquired immunodeficiency syndrome among healthy population	Jain M. [30]	2020	Human immunodeficiency virus (HIV)-related stigma is present at all levels, which act as critical barriers for effectively addressing it. This also influences the treatment uptake and under- or non-participation in treatment available. In view of this, the present study was aimed to assess the stigma of otherwise healthy individuals of the community toward HIV infection/acquired immunodeficiency syndrome (AIDS). The study was conducted on 100 healthy individuals. The mean age of the participants was 36.99 ± 12.38 years. Approximately half of the participants were below the age of 35 years. The majority (77%) of the participants were males and 70% of the total participants were educated above the intermediate level (12th standard). The mean years of education of the participant group with education above 12th standard (n = 70) was 15.99 (standard deviation [SD] = 1.21). Participants with education ≤12th standard (n = 30) had 10.33 (SD = 1.18) mean years of education. The difference in the years of education between these two groups was statistically significant (*p* < 0.0001). Participants were divided into two groups on the basis of their level of education (<12 years of formal education and >12 years of formal education). Stigma related to HIV/AIDS was compared between these two groups, and there was no significant difference in the level of stigma in these distinctly different educational groups. Their responses were taken on a self-designed semi-structured questionnaire. The results showed that there is more perceived stigma as compared to enacted stigma. Nearly 46% of the individuals feel that HIV-infected persons should be blamed for their illness and 41% individuals feel that they will feel ashamed if they have HIV. It was also seen that older adults (between 46 and 55 years) had more stigma as compared to the younger adults (between 16 and 25 years). The educated individuals still have stigma to a certain extent. Most of the individuals would like to tell their partners if they were diagnosed with HIV. This study shows how, today, there is still stigma present, to a certain extent, in society, in educated and urban individuals. The level of stigma may not be significantly different in people with educational differences. Stigma needs to be addressed for prevention and better management of HIV/AIDS.
Who is the bigger stigmatizor? The loved one or the society	Yildirim Z. [31]	2019	Epilepsy has long been considered by society as a dangerous and frightening spiritual possession or even a contagious disease. The aim of this study was to determine the stigmatizing level of the general Turkish population and to compare these results with the stigmatizing level of the patients’ relatives group (PRG). This study used two scales for the quantification of the stigma level in patients with epilepsy (PWE) and their relatives. Sample: 202 healthy people who were caregivers and relatives of neurologic patients, other than epilepsy, were recruited for the control group (CG). A sociodemographic and clinical data form and Stigma Scale for Epilepsy (SSE)-Informant Report (IR) scale were administered to the CG as well. The relationship between sociodemographic characteristics and SSE-IR scores was evaluated, and a regression analysis was performed in order to analyze sociodemographic factors contributing to SSE-IR scores. Stigmatizing levels were compared between PRG and CG. Results: comparison of stigma scores among different socio-demographic strata of the CG showed that there was a statistically significant difference in terms of educational status and occupation (*p* < 0.01). Multivariate linear regression analysis revealed that education accounted for 10.8% and 8.9% of the variance in the SSE-IR scale, respectively, in the PRG and the CG. Prejudgment scores and total scores of the PRG were significantly higher than those of the CG. There was no statistically significant difference between two groups in terms of discrimination and false beliefs subscales scores. The proportion of highly stigmatized participants in the PRG was statistically significantly higher than that of the CG. This study showed us that the stigmatization levels in a group of subjects drawn from general population without acquaintance of epilepsy were lower than the relatives of the patients. This result may be partially explained by the ambivalent attitudes of the relatives, as those high scores may stem from, not only enacted, but also the felt stigma that they were experiencing themselves. It must be a warning sign for all of the clinicians treating epilepsy, and the national association against epilepsy, as well as public health officials, to increase efforts for awareness raising.
Experiences of patients with primary HIV diagnosis in Kermanshah-Iran regarding the nature of HIV/AIDS: A qualitative study	Barkish F.G. [32]	2019	This study aimed to examine the attitude, understanding, and interpretation of a positive diagnosis of HIV. Participants included 13 HIV-positive patients who had been referred to the Kermanshah Counseling Center for Behavioral Diseases in Iran, and diagnosed at most 6 months before being interviewed. The data were collected via semi-structured in-depth interviews. Of the 13 participants in this study, 47% (n 1⁄4 6) were male. Participants had received their diagnosis 2–6 months before being interviewed; the mean time of the diagnosis was 4.8 months. None of the participants received antiretroviral therapy. The continuous analyses of the data and the interview notes resulted in the identification of five main themes: contagious disease with two sub themes (illness and harm to others), new self with an identity subtheme, disappointment with the life ending, and impending death subtheme, unmentionable disease with two sub themes (secretive and horrifying diseases), and loss with frustration subtheme. Because the attitudes and behaviors of other people (such as relatives and people in society) had a negative impact on the participants’ mental status, as well as on their perceptions and understanding about the positive diagnosis, the focus of care for people with HIV/AIDS should shift from therapeutic issues to socio-cultural ones.
Validation of the Perceived Stigmatization Questionnaire for Brazilian adult burn patients	De Oliveira Freitas N. [33]	2018	Currently, there is no questionnaire to assess perceived stigmatization among people with visible differences in Brazil. The Perceived Stigmatization Questionnaire (PSQ), developed in the United States, is a valid instrument to assess the perception of stigmatizing behaviors among burn survivors. The objective of this study was to assess the factor structure, reliability, and validity of the Brazilian Portuguese version of the PSQ in 240 burn patients. Internal consistency was assessed by calculating Cronbach’s alpha for the PSQ total score and subscales, and for domains of Burn Specific Health Scale-Revised (BSHS-R), Self-Esteem Scale (RSES) and the Beck Depression Inventory (BDI). Reproducibility of the PSQ was evaluated with a test-retest design and computed using as the two-way mixed effects intraclass correlation coefficient (ICC). For Cronbach’s alpha and the ICC, values greater than 0.70 were considered acceptable. The distribution of PSQ item responses was calculated in order to assess floor and ceiling effects. A floor or ceiling effect was considered to occur when more than 15% of the participants attained the lowest (floor) or highest (ceiling) possible score. Item total and item subscale score correlations were analyzed and considered as adequate values between 0.20 and 0.70. Values below 0.20 should be discarded.
Stigma and Ebola survivorship in Liberia: Results from a longitudinal cohort study	Overholt [34]	2018	Survivors of the 2014–2016 West Africa Ebola epidemic have been reported to suffer high levels of stigmatization after return to their communities. They found, through the study of 299 participants, that Ebola-related stigma was prevalent among Liberian survivors more than a year after Ebola Virus Disease (EVD) recovery. Self-reported stigma was greater in the period before cohort enrollment; however, some degree of stigmatization persisted years after EVD. Transient rises in stigma were observed following episodic Ebola re-emergence of EVD in Liberia. The median time from Ebola Virus Disease (EVD) to study entry was 393 days (IQR—interquartile range—336–492). Participants (43% female) had a median age of 31 (IQR 25–40) years. Mean self- reported stigma levels were greater at baseline (6.28 ± 0.15 (IQR: 4.38–8.75)) compared to the first post-baseline visit (0.60 ± 0.10 (IQR: 0–0); *p* < 0.0001). During follow-up, stigma levels were stable. Baseline stigma significantly increased during enrollment and following clusters of Ebola re-emergence in Liberia. Survivors encountered primarily enacted and perceived external stigma rather than internalized stigma.
A prospective study on Quality of Life in Patients with Pulmonary Tuberculosis at a Tertiary Care hospital in Kashmir, Northern India	Dar S.A. [35]	2018	Pulmonary Tuberculosis (PTB) is a contagious, airborne infection that destroys when *Mycobacterium tuberculosis* primarily attacks the lungs. Pulmonary tuberculosis is curable with an early diagnosis and antibiotic treatment. Stigmatization and negative emotions resulting from the illness could result in long-term impairment of patients’ psychological well-being, which may result in work absenteeism resulting in loss of productivity and reduced monthly income. This was a prospective study, which was conducted over a period of one-and-a-half years. A total of 198 patients were recruited for the study. Quality of life was assessed at baseline and at the end of the intensive phase. For quality of life, World Health Organization Quality of Life Instruments (WHO QOL-BREF) was used. In the present study, patients scored lowest in the baseline physical (8.36 ± 1.60) followed by the psychological domain (10.40 ± 1.72); however, at the end of the intensive phase, both physical (11.98 ± 1.70) and psychological (12.75 ± 1.) domains improved very much, and the difference was statistically significant. They conclude that Health Related Quality of Life (HRQOL) is significantly reduced in patients with PTB, and that it improves rapidly and significantly with Directly Observed Treatments (DOTS)-based intensive phase of treatment. Special focus on reduction of stigmatization should be given in the management of TB to reduce the psychological distress.
Chronic mass psychogenic illness among women in Derashe Woreda, Segen Area People Zone, southern Ethiopia: a community based cross-sectional study	Ayehu M. [36]	2018	The purpose of this study is to show the outbreaks of mass psychogenic illness (MPI), which are a constellation of physical signs and symptoms suggestive of organic illness with no identifiable causes. MPI has been documented in numerous cultural, ethnic, and religious groups throughout the world. The aims of this study was to document the nature and impact of the illness, to assess interventions, and to come up with recommendations and management formulations for dealing with such kinds of outbreaks in the future. A community based cross-sectional study was conducted in June 2015 in Dirashe, a woreda in the Segen Area Peoples Zone of the Southern Nations, Nationalities, and Peoples’ Region of Ethiopia. Women with complaints of breast cancer, but with no objective findings, were the subjects of the study. Ninety-seven women were investigated using a semi-structured questionnaire for quantitative study. Two focus group discussions with seven affected and seven non-affected women, and four key informant interviews, were conducted using guiding questionnaires. Quantitative data were analyzed using SPSS version 20 software packages while qualitative data was analyzed manually going through thematic areas. The ages of the ninety-seven study participants ranged from 17 to 56 years, with a mean (SD) of 32.8 (8.7) years. Onset of illness dated back to the year 2012 following the death of 43 socially active women with complications of breast cancer. Following their death, many women started to report multiple vague physical complaints similar to those of the deceased woman. Even though the responses from the study participants did not specifically point to a single possible cause and means of transmission, high numbers of women believed the source of their illness could be punishment from God, while some said that the cause of their suffering could be environmental pollution. Since the illness was taken to be contagious, affected women faced stigma and discrimination. Moreover, school activities and social gatherings were limited significantly. Unrealistic and exaggerated rumors and inadequate explanations about the nature and spread of the illness were the main contributing factors for the spread and prolongation of the outbreak. An organized intervention, clear and adequate explanations about the nature and transmission of the illness, can contain MPI within a short period of time.
A survey of the knowledge, attitudes and practices on Zika virus in New York City	Samuel G. [22]	2018	This study shows over 900 travel-associated Zika virus cases have been identified in New York City (NYC), New York. A survey was administered in NYC adapted from the knowledge, attitudes, and practices (KAP) survey on the Zika virus developed by the World Health Organization (WHO). A standardized, self-administered, anonymous questionnaire was administered to a convenience sample in Manhattan and the Bronx from 30 June, 2016 to 21 October, 2016. Responses were grouped into six domains based on the content and structure of the questions, and were summarized using descriptive statistics or converted into a continuous knowledge score and assessed for associations with pregnancy status and travel history using linear regression. There were 224 respondents with a mean age of 33 (SD ± 11.6); 77% (170/224) were female and 24% (51/224) pregnant. The majority (98% (213/217)) were unable to identify all of the symptoms associated with acute Zika virus infection and all modes of transmission (97% (213/219)). Most participants (85% (187/219)) identified mosquitoes as a mode of transmission. Moreover, 95% (116/122) reported an association between Zika virus and microcephaly. The most concerning aspect of Zika virus in 46% (91/200) was the risk of disabilities to babies, and risk of sexual transmission (25% (49/200)). When asked what precautions pregnant persons should take to reduce the risk of transmission when traveling to a Zika endemic region, only 27% (50/185) identified using condoms during intercourse or refraining from intercourse while pregnant. Knowledge of Zika transmission is significantly positively associated with pregnancy status, but not with travel history. These results indicate an overall poor understanding of Zika virus symptoms and possible complications, transmission modes, and current recommended prevention guidelines. Pregnancy is positively associated with Knowledge of Zika Transmission, but not other knowledge scores. Reported travel history to Zika endemic regions is not significantly associated with Zika knowledge. There is a need for implementing future public health interventions that particularly focus on protection against Zika transmission—that Zika is sexually transmitted, and risks that the Guillain-Barré Syndrome poses a risk to adults.
Assessment of stigma among patients infected with hepatitis C virus in Suez City, Egypt	Soltan E.M. [37]	2017	This study aimed to assess the presence of stigma in patients with chronic hepatitis C, and to assess the relationship between socio-demographic characteristics and stigma. This is a cross-sectional descriptive and analytic study. This study was carried out at the communicable diseases, research and training center affiliated with Suez Canal University, Suez Governorate, Egypt. The sample included 260 patients with hepatitis C who filled in a questionnaire asking about the socio-demographic characteristics and hepatitis C stigma scale. There was at least one stigmatizing characteristic in 155 (59.6%) of the patients with HCV. Among them, 53 (20.4%) reported that he/she was not the same as the others, 54 (20.8%) felt dirty, and 112 (43.1%) felt that he/she was a bad person. Participants also agreed that people with hepatitis C are repulsive and rejected, according to 55 (21.2%) and 64 (24.6%), respectively. Sixty-seven (25.8%) had been hurt by the reactions of others. Among them, 52 (20%) stopped hanging out with others because of their reactions, 53 (20.4%) lost friends, and 55 (21.2%) were worried that others would tell about their illness. The marital relationship was affected by the diagnosis of hepatitis in 134 (51.5%) participants. Subjects who were younger and who were married had higher stigma scores (*p* = 0.018, 0.013). Smokers were more rejected (*p* = 0.007) and hurt by reactions of others than non-smoking patients (*p* = 0.013); they had lost more friends (*p* = 0.002) and were more worried that others would tell about their illness (*p* = 0.016). Patients with hepatitis C feel stigmatized in different areas; there is a need for implementation of educational programs to raise the awareness of community and healthcare providers about the stigma of hepatitis C and its negative consequences, to act as advocates for their patients.
Effect of Stigma and Concealment on Avoidant-Oriented Friendship Goals	Lattanner M.R. [28]	2017	In this research, they propose a hypothesized model that outlines pathways by which stigma impacts interpersonal behavior within close relationships through avoidant-oriented friendship goals. They also examine how stigma concealment moderates the extent to which these avoidant goals are activated. In Study 1, included 120 participants who were on average 32.5 years old, with a mental illness, and currently experiencing a mental health problem(s). Among people with mental illness (PWMI), the relationship between internalized stigma and self-silencing was mediated by avoidant-oriented friendship goals. The mean score for levels of internalized stigma fell slightly below the midpoint (M = 2.53, SD = 1.18). Consistent with past social goals research (Murray, Derrick, Leader, & Holmes, 2008), participants more strongly endorsed approach goals (M = 4.62, SD = 1.24) as compared to avoidant oriented goals (M = 4.09, SD = 1.51). In addition, participant responses on the measure of concealment (M = 2.83, SD = 1.08). In Study 2, there was a community sample of 240 participants (18 years of age or older with a mental illness and currently experiencing mental health problems). Stigmatized identity salient increased the endorsement of avoidant-oriented friendship goals, particularly for people relatively high in concealment. Collectively, these studies highlight a social dilemma encountered by PWMI; what may be adaptive regulatory responses to stigmatization can motivate behavior that has negative effects in close relationships. Participants in the stigma condition endorsed avoidant oriented goals (M = 4.01, SD = 1.36) at a higher level compared to participants in the control condition (M = 3.61, SD = 1.46) and endorsed approach-oriented friendship goals at a lower level (M = 4.64, SD = 1.32) than participants in the control condition (M = 4.8, SD = 1.25). Average level of concealment fell near the midpoint indicating that it was somewhat true that participants were concealing from friends (M = 2.99, SD = 1.04). There was no evidence that level of concealment was confounded by manipulation as average level of concealment did not statistically differ across conditions (M_stigma_ = 3.07, SD = 1.10; M_control_ = 2.91, SD = 0.99, t(185) = −1.05, *p* = 0.30). Similar to Study 1, there were no significant differences in avoidant-goals, approach-goals, or concealment across diagnostic criteria, treatment type, ethnicity, relationship status, or gender, *p* > 0.05.
Measuring HIV- and TB-related stigma among healthcare workers in South Africa: a validation and reliability study	Wouters E. [38]	2017	Recent evidence indicates that human immunodeficiency virus (HIV) and tuberculosis (TB) related stigma act as key barriers to the utilization of associated occupational health services by South African healthcare workers (HCWs). It also highlights a dearth of appropriate tools to measure HIV and TB stigma among HCWs. The current study employs data from a study on HIV and TB stigma among HCWs, a cluster randomized controlled trial for the collection of data among 882 HCWs in the Free State Province of South Africa. Four test scales measuring different aspects of stigma were used: respondent’s external stigma (RES) and others’ external stigma (OES) towards TB as well as HIV across different professional categories of HCWs. The analytical strategy consisted of the following four steps: (1) confirmatory factor analysis (CFA) testing for internal construct validity, (2) reliability testing (Cronbach’s a), (3) testing of configural, metric and scalar invariance across the two subgroups (patient staff and support staff) using differences in v2, and (4) structural equation modeling to test for external construct validity (correlations between stigma scales and with TB and HIV knowledge and confidentiality). All analyses were performed using Mplus v7.4 (Muthén & Muthén, Los Angeles, CA, USA). Confirmatory factor analyses and structural equation modeling were used to assess the validity and reliability of the scales. All four scales displayed adequate internal construct validity. Subsequent analysis demonstrated that all four scales were metric-invariant, and that the OES scales were even scalar-invariant across patient and support staff groups. The scales displayed good reliability and external construct validity. The results support the use of the scales developed to measure TB and HIV stigma among HCWs. Further research is, however, needed to fine-tune the instruments and test them across different resource-limited countries.
The Development and Piloting of Parallel Scales Measuring External and Internal HIV and Tuberculosis Stigma Among Healthcare Workers in the Free State Province, South Africa	Wouters E. [39]	2016	The dual burden of tuberculosis and human immunodeficiency virus (HIV) is severely impacting the South African healthcare workforce. However, the use of on-site occupational health services is hampered by stigma among the healthcare workforce. The success of stigma-reduction interventions is difficult to evaluate because of a dearth of appropriate scientific tools to measure stigma in this specific professional setting. The current pilot study aimed to develop and test a range of scales measuring different aspects of stigma—internal and external stigma toward tuberculosis as well as HIV—in a South African healthcare setting. The study employed data of a sample of 200 staff members of a large hospital in Bloemfontein, South Africa. Confirmatory factor analysis produced seven scales, displaying internal construct validity: (1) colleagues’ external HIV stigma, (2) colleagues’ actions against external HIV stigma, (3) respondent’s external HIV stigma, (4) respondent’s internal HIV stigma, (5) colleagues’ external tuberculosis stigma, (6) respondent’s external tuberculosis stigma, and (7) respondent’s internal tuberculosis stigma. Subsequent analyses (reliability analysis, structural equation modeling) demonstrated that the scales displayed good psychometric properties in terms of reliability and external construct validity. The study outcomes support the use of the developed scales as a valid and reliable means to measure levels of tuberculosis- and HIV-related stigma among the healthcare workforce in a resource-limited context. Future studies should build on these findings to fine-tune.
Psychological Distress among Ebola survivors Discharged from an Ebola Treatment Unit in Monrovia, Liberia—a Qualitative study	Rabelo I. [40]	2016	A consequence of the West Africa Ebola outbreak from 2014–2015 was the unprecedented number of Ebola survivors discharged from the Ebola Treatment Units (ETUs). Liberia alone counted over 5000 survivors. In this study, they undertook qualitative work in Monrovia to better understand the mental distress experienced by survivors during hospitalization and reintegration into their community. Purposively selected Ebola survivors from ELWA3 (Ebola Case Management Center run by MSF in Monrovia), the largest ETU in Liberia, were invited to join focus group discussions. Three focus groups with a total of 17 participants were conducted between February and April 2015. To favor participation, groups were divided by gender. The first group included nine female survivors and was conducted in a tent used for psychosocial group counseling, outside the premises of the ETU. The second and third focus group discussions (FGDs) included male EVD survivors, and were conducted inside the survivor clinic. The second FGD included eight male survivors, of whom six were invited to a third FGD. An interview guide with open-ended questions was applied. There were four main themes: (1) mental health distress during treatment at ELWA3, (2) coping strategies to overcome mental health distress in ELWA3, (3) mental health distress after discharge from ELWA3, and (4) coping strategies after discharge from ELWA3. It has been noted that exposure to death in the ETU and stigma in the communities induced posttraumatic stress reactions and symptoms of depression among Ebola survivors. Distress in the ETU can be reduced through timely management of corpses. Coping mechanisms can be strengthened through trust relationships, religion, peer/community support, and community-based psychosocial care. Mental health disorders need to be addressed with appropriate specialized care and follow-up.
Knowledge and attitude of key community members towards tuberculosis: mixed method study from BRAC TB control areas in Bangladesh	Paul S. [41]	2015	Bangladesh National Tuberculosis (TB) Control Program adopted a number of strategies to facilitate TB diagnosis and treatment. ‘Advocacy, Communication and Social Mobilization’ (ACSM) was one of the key strategies implemented by the Bangladesh Rural Advancement Committee (BRAC), a non-governmental development organization TB control program. The purpose of this study is to assess the knowledge and attitudes of the key community members (KCMs) participated in ACSM in BRAC TB control areas. This study combined quantitative and qualitative methods using a mixed method approach. KCMs in three districts with low TB case detection rates were targeted to assess the ACSM program. The quantitative survey using a multi-stage random-sampling strategy was conducted among 432 participants. The qualitative study included in-depth interviews (IDIs) of a subsample of 48 respondents. For quantitative analysis, descriptive statistics were reported using frequencies, percentages, and Chi square tests, while thematic analysis was used for qualitative part. The quantitative survey questionnaire was administered by 36 enumerators under the guidance of six research assistants supervised by the first and second author. Prior to data collection, a one-day extensive training session was conducted, which included a background briefing on the project and its objective. The training also disseminated information on ACSM activities undertaken by the BRAC TB control program. The questionnaire was prepared in English and translated into Bengali (local language). After pre-testing, the Bengali version of the questionnaire was modified and back translated into English, incorporating the feedback from the pre-testing. All of the instructions, including skipping and probing, were documented as a field protocol to assist the field workers. Six qualitative researchers, along with two other authors, were involved in qualitative data collection. Responses to the IDIs were made both through notes during interviews and audio recordings. Most (99%) of the participants had heard about TB, and almost all knew that TB is a contagious yet curable disease. More than half (53%) of the KCMs had good knowledge regarding TB, but BRAC workers were found to be more knowledgeable compared to other KCMs. However, considerable knowledge gaps were observed among BRAC community health workers. Qualitative results revealed that the majority of the KCMs were aware about the signs, symptoms, and transmission pathways of TB and believed that smoking and addiction were the prime causes of transmission of TB. The knowledge about child TB was poor, even among BRAC health workers. Stigma associated with TB was not uncommon. Almost all respondents expressed that young girls diagnosed with TB had not enough knowledge and did not really understand the mode of transmission and the risks of the disease. This study finding has revealed varying levels of knowledge and mixed attitudes about TB among the KCMs. It also provides insight on the poor knowledge regarding child TB and indicates that, despite the significant success of the TB program, stigma is yet prevalent in the community. Future ACSM activities should engage community members against stigma and promote child TB-related information for further improvement of the BRAC TB Control Program.
Investigation of perceived stigma among people living with human immunodeficiency virus/acquired immune deficiency syndrome in Henan Province, China	Li Z. [26]	2014	The purpose of this study is to investigate the level of, and factors influencing, perceived stigma and discrimination among people living with human immunodeficiency virus (HIV)/acquired immune deficiency syndrome (PLWHA) in Henan Province. In total, 161 PLWHA from Zhengzhou and Zhenping were investigated using the Berger HIV stigma scale. The mean Berger stigma scale score was 105.70 ± 15.20, indicating a middle stigma level. Among the four subscales of the Berger stigma scale, the disclosure concerns score was highest, while the negative self-image score was lowest. Multivariate analyses showed that factors influencing perceived HIV stigma included the level of education and route of infection. The level of perceived HIV stigma and discrimination among PLWHA in Henan Province is moderate and was affected by the level of education and route of infection.
Contagiousness under antiretroviral therapy and stigmatization toward people with HIV	Drewes J. [42]	2014	Perceived contagiousness is a major dimension underlying HIV-related stigmatization. Antiretroviral therapy (ART) can diminish contagiousness by reducing viral load levels in HIV-infected individuals. To test the assumption that reductions in contagiousness can lead to a decrease in stigmatizing reactions, they conducted an experimental online study. A sample of 752 participants (50.9% female) read a short vignette depicting HIV-positive individuals with either high or low viral loads, and were either given, or not given, information about the association between viral load and contagiousness. Subsequently, participants were asked to rate their willingness to stigmatize this individual by responding to two measures of social and physical distance. Differences between the low and the high viral load information groups and the combined no-information groups (forming a quasi-control group) were analyzed using analysis of covariance (ANCOVA), controlling for gender and baseline perceptions of contagiousness. The covariates perceived contagiousness at baseline and gender were associated with social and physical distancing, but the viral load/information factor was only significant in physical distancing. Planned contrast analyses confirmed that physical distancing in the informed group was lower in the low viral load condition compared to the high viral load condition, and to the control group. They found evidence for the significant role of perceived contagiousness in the HIV-related stigma and were able to experimentally demonstrate the potential of ART to reduce HIV-related stigmatization by lowering viral load and contagiousness, when these changes are accompanied by a decreased perception of contagiousness.
The Acceptance and Action Questionnaire–Stigma (AAQ-S): Developing a measure of psychological flexibility with stigmatizing thoughts	Levin M.E. [43]	2014	The current study sought to develop and test the Acceptance and Action Questionnaire—Stigma (AAQ-S), a measure of psychological flexibility with stigmatizing thoughts. The study used a sample of 604 undergraduate students participating in an online survey for extra credit in a psychology course at the University of Nevada, Reno. The sample was 67.7% female and the modal age was 18 (M = 20.30, Standard Deviation (SD) = 3.93). The racial distribution of the sample was 70.2% White, 9.8% Asian, 4.6% Black or African American, 1.7% Native Hawaiian/Other Pacific Islander, 1.7% Native American, 7.1% other and 4.9% Multiracial. In addition, 14.8% described their ethnicity as Hispanic or Latino. Assessments were completed anonymously, and participants had the option to skip any question using the option “I choose not to answer.” Ethical approval for the study was provided by the University of Nevada, Reno Internal Review Board. With each participant, mean scores were calculated for each variable, provided that 80% of the items were answered on a given scale or subscale. If a participant answered fewer than 80% of the items on a scale or subscale, then a sum score was not calculated for that variable. Based on these criteria, between 0% and 4% of participants were missing scale scores for specific variables (the social distance scale was the most common missing variable resulting from the 80% cutoff). Expert judge ratings and factor analyses were used to identify and refine two distinct subscales: psychological flexibility and psychological inflexibility, relating to stigmatizing thoughts. Analyses indicated that the AAQ-S psychological flexibility and inflexibility subscales, as well as a combined total score, correlate with other measures of psychological flexibility and stigma in expected ways, and are more predictive of stigma than a general measure of psychological flexibility. Overall, the results suggest that the AAQ-S could be a useful measure in conducting future stigma research.
Barriers in the Management of Tuberculosis in Rawalpindi, Pakistan: A Qualitative Study	Soomro M.H. [44]	2013	Tuberculosis (TB) is a contagious, airborne disease and remains a major global public health hazard. TB is a major cause of mortality and is affecting millions of people in low-income and middle-income countries. Worldwide, one person out of three is infected with *Mycobacterium tuberculosis*. Timely diagnosis and treatment are the two key factors for better outcomes. Non-adherence to TB treatment is an important barrier for TB control programs. This study was designed to understand the barriers encountered by TB patients when seeking healthcare. A random sample of 30 TB patients was selected for in- depth interviews after grouping them, according to their type of treatment outcome. In order to select patients for the in-depth interviews, health facility registers (TB registers TB03) were used. These patients were then approached, and finally, 23 patients (13 males and 10 females) aged 15–65 years were interviewed. Four patients from the unfavorable group (including failure and defaulted cases) were not accessible; the address of one patient was wrong while three patients refused to give interviews. The addresses of three patients were wrong among the treatment success group (cured and treatment success cases). There were 15 participants from urban and eight from rural settings. In addition, 15 in-depth interviews were also conducted with DOTS facilitators (five from urban settings, and 10 from rural settings); four participants were females from rural settings, while 11 participants were males (six from rural and five from urban settings). The age range was between 21 and 47 yrs., with 1–8 years of experience as a DOTS facilitator. The majority of them had two years of experience. All participants had attended the training course on TB DOTS paramedic’s module. The entire interviews were recorded in writing, which were then translated to English, transcribed verbatim, coded, and categorized into main themes. The study was approved by the National Bioethical Committee (NBC) of Pakistan. Confidentiality was ensured by not disclosing the identity of participants, and written informed consent was obtained from all patients. Most patients were found to be well informed about the idea of taking TB medications under direct supervision and its overall importance. However, many of them were not convinced with either drugs or treatment protocols. It was found that limited knowledge of patients, loss of employment, financial burden, social stigma, and long distance from health facilities were the main barriers for TB adherence. More patient-centered interventions and attention to the barriers are required to improve the treatment adherence. Direct observation of patients and regular home visits by health workers can reduce the risk of non- adherence.
Professional nurses’ views regarding stigma and discrimination in the care of HIV and AIDS patients in rural hospitals of the Limpopo province, South Africa	Manganye B.S. [45]	2013	The aim of the study was to determine the views of professional nurses on the manifestations of HIV and AIDS stigma and discrimination and their influence on the quality of care rendered to people living with HIV and AIDS in three rural hospitals of Limpopo province, South Africa. The study was qualitative, exploratory, descriptive, and contextual in nature. The population included all professional nurses registered with the South African Nursing Council who were working with confirmed HIV-positive patients in the three hospitals and had received specialized training in voluntary counseling and testing (VCT), antiretrovirals (ARVs), prevention of mother-to-child transmission (PMTCT), and couples counseling. A purposive sampling method was used to select both the wards and participants, based on set criteria. A total of nine wards (six adult medical and three maternity) and 37 participants were selected. Focus group discussions and semi-structured and key informant interviews were conducted. Data were analyzed using a combination of data analysis guidelines from different sources. A total of 185 (39.87%) of the professional nurses were trained in one or more HIV and AIDS related courses. The total 221 (47.63%) indicates the number of individuals who had attended different courses in the different hospitals. Results revealed that professional nurses were aware of the existence of HIV and AIDS stigma and discrimination in their wards and regarded these as bad and improper care of HIV-positive patients. Behavior included leaving care of HIV patients to junior members of staff with limited skills and knowledge of HIV and AIDS; showing HIV-positive patients that their disease was dangerous and contagious; judgmental behavior towards and stereotyping of HIV-positive patients; and regarding patients with HIV and AIDS as uncooperative and problematic in the wards.
The development and validation of the Questionnaire on Anticipated Discrimination (QUAD)	Gabbidon J. [46]	2013	The anticipation of mental health-related discrimination is common amongst people with mental health problems and can have serious adverse effects. This study aimed to develop and validate a measure assessing the extent to which people with mental health problems anticipate that they will personally experience discrimination across a range of contexts. The items and format for the Questionnaire on Anticipated Discrimination (QUAD) were developed from previous versions of the Discrimination and Stigma Scale (DISC), focus groups, and cognitive debriefing interviews, which were used to further refine the content and format. The resulting provisional version of the QUAD was completed by 117 service users in an online survey and reliability, validity, precision, and acceptability were assessed. A final version of the scale was agreed and analyses re-run using the online survey data and data from an independent sample to report the psychometric properties of the finalized scale. The provisional version of the QUAD had 17 items, good internal consistency (alpha = 0.86) and adequate convergent validity as supported by the significant positive correlations with the Stigma Scale (SS) (r = 0.40, *p* < 0.001) and the Internalized Stigma of Mental Illness Scale (ISMI) (r = 0.40, *p* < 0.001). Three items were removed due to low endorsements, high inter-correlation or conceptual concerns. The finalized 14 item QUAD had good internal consistency (alpha = 0.86), good test retest reliability (ρc = 0.81) and adequate convergent validity: correlations with the ISMI (r = 0.45, *p* < 0.001) and with the SS (r = 0.39, *p* < 0.001). Reading ease scores indicated good acceptability for general adult populations. Cross-replication in an independent sample further indicated good internal consistency (alpha = 0.88), adequate convergent validity, and revealed two factors summarized by institutions/services and interpersonal/professional relationships. The QUAD expanded upon previous versions of the DISC. It is a reliable, valid, and acceptable measure that can be used to identify key life areas in which people may personally anticipate discrimination, and an overall tendency to anticipate discrimination. It may also be useful in planning interventions aimed at reducing the stigma of mental illness.
Is participation contagious? Evidence from a household vector control campaign in urban Peru	Buttenheim A.M. [47]	2013	High rates of household participation are critical to the success of door-to-door vector control campaigns. The purpose of this paper is to use the Health Belief Model to assess determinants of participation, including neighbor participation as a cue to action, in a Chagas disease vector control campaign in Peru. In this study, they evaluated clustering of participation among neighbors; estimated participation as a function of household infestation status, neighborhood type, and number of participating neighbors; and described the reported reasons for refusal to participate in a district of 2911 households. They observed significant clustering of participation along city blocks (*p* < 0.0001). Participation in the first round of the attack phase in Mariano Melgar (MM), district of Arequipa, was 66% (6336 of 9579 total properties). They observed geographical clustering of participation, including significant runs of participation along blocks (*p* < 0.0001, Siegel–Castellan Runs Test). While this test of spatial autocorrelation does not imply that neighbor behavior influences participation decisions, it does suggest some social contagion. Participation in the transect sample was 77% (340 of 433 total properties), higher than in the MM overall. However, there were stark differences in participation rates along the transect. Most notably, participation was higher in new versus established neighborhoods and for infested versus non-infested households. Furthermore, the upward slope for the non-infested households in new neighborhoods indicates an association between the number of neighbors participating and household participation in this subgroup: the probability of participation was 50% for households with no neighbors participating versus 92% for households with both adjacent neighbors participating. Participation was significantly higher for households in new versus established neighborhoods, for infested households, and for households with more participating neighbors. The effect of neighbor participation was greater in new neighborhoods. Results support a ‘contagion’ model of participation, highlighting the possibility that one or two participating households can tip a block towards full participation. Future campaigns can leverage these findings by making participation more visible, by addressing stigma associated with spraying, and by employing group incentives to spray.
Validation of an HIV-related stigma scale among healthcare providers in a resource-poor Ethiopian setting	Feyissa G.T. [48]	2012	Stigma and discrimination (SAD) against people living with human immunodeficiency virus (HIV) are barriers affecting effective responses to HIV. Understanding the causes and extent of SAD requires the use of a psychometrically reliable and valid scale. The objective of this study was to validate an HIV-related stigma scale among healthcare providers in a resource-poor setting. A cross-sectional validation study was conducted in 18 healthcare institutions in southwest Ethiopia, from 14 March, 2011 to 14 April, 2011. A total of 255 healthcare providers responded to questionnaires asking about sociodemographic characteristics, HIV knowledge, perceived institutional support (PIS), and HIV-related SAD. Exploratory factor analysis (EFA) with principal component extraction and varimax with Kaiser normalization rotation were employed to develop scales for SAD. Eigenvalues greater than 1 were used as a criterion of extraction. Items with item-factor loadings less than 0.4 and items loading onto more than one factor were dropped. The convergent validity of the scales was tested by assessing the association with HIV knowledge, PIS, training on topics related to SAD, educational status, HIV caseload, presence of an antiretroviral therapy (ART) service in the healthcare facility, and perceived religiosity. Seven factors emerged from the four dimensions of SAD during the EFA. The factor loadings of the items ranged from 0.58 to 0.93. Cronbach’s alphas of the scales ranged from 0.80 to 0.95. An in-depth knowledge of HIV, perceptions of institutional support, attendance of training on topics related to SAD, degree or higher education levels, high HIV caseloads, the availability of ART in the healthcare facility, and claiming oneself as nonreligious were all negatively associated with SAD, as measured by the seven newly identified latent factors. The findings in this study demonstrate that the HIV-related stigma scale is valid and reliable when used in resource-poor settings. Considering the local situation, healthcare managers and researchers may use this scale to measure and characterize HIV-related SAD among healthcare providers. Tailoring for local regions may require further development of the tool.
Disease-avoidant behaviour and its consequences	Kouznetsova D. [19]	2011	Medical conditions that are non-contagious, but that appear contagious, seem to result in the sufferer being avoided. Error management theory (EMT), suggests that such false alarms occur because the cost of infection poses a greater threat to one’s fitness than avoidance. Study 1 attempted to demonstrate a disease-related false alarm effect by asking participants, to evaluate a series of vignettes, featuring people with infectious diseases, non- infectious diseases that looked infectious and non-infectious diseases that did not. Judgements of contracting infection under varying levels of contact, and desire to avoid were obtained. Consistent with EMT, a false alarm effect was evident. Study 2 examined the importance of the face as a key indicator of real and apparent infection, by determining whether facial symptoms result in a greater desire to avoid people with infectious and non- infectious diseases. Consistent with expectation, participants reported a greater desire to avoid people with facially displayed symptoms. Together, these results support the idea that humans have evolved a general tendency to avoid individuals with disease signs, especially if displayed upon the face. One consequence is that where a facially displayed disease sign persists, even if known to be benign, its bearer will experience chronic avoidance.
Tuberculosis knowledge, attitudes and health-seeking behaviour in rural Uganda	Buregyeya E. [49]	2011	The purpose of this study is to assess tuberculosis (TB) knowledge, attitudes, and health-seeking behavior to inform the design of communication and social mobilization interventions. Between June and July 2008, in Iganga/Mayuge Demographic Surveillance Site, Uganda, focus group discussions (FGDs) and key informant interviews (KIs) were conducted among both male and female parents/caretakers of children and adolescents, school heads, opinion leaders and TB patients. Eighteen FGDs were conducted, including six FGDs of young mothers/fathers/caretakers (aged <36 years) of children, six of mothers/fathers/caretakers of adolescents, and six of mature mothers/fathers/caretakers aged ≥36 years). Key informants included two local council leaders (LCs) and two traditional healers, known as mukalakasa. Mukalakasa are men or women who provide herbal and/or spiritual healing. Interviewees also included two TB patients, two religious leaders (a Muslim and a Christian), two elders, and two sub-county TB supervisors (health assistants). FGDs and KIs were conducted at the village level. A purposive sampling method (with the help of the LCs) was used to identify participants for the FGDs and KIs. FGDS were grouped by such factors as age and sex, as homogeneity of focus group participants can facilitate sharing. Eighteen FGDs were conducted, including six FGDs of young mothers/fathers/caretakers (aged <36 years). It has been noted that people viewed TB as contagious, but not necessarily an airborne pathogen. Popular TB etiologies included sharing utensils, heavy labor, smoking, bewitchment, and hereditary transmission. TB patients were perceived to seek care late or to avoid care. Combining care from traditional healers and the biomedical system was common. Poverty, drug stock-outs, fear of human immunodeficiency virus (HIV) testing, and length of TB treatment negatively affect health-seeking behavior. Stigma and avoidance of persons with TB often rejects an assumption of HIV co-infection. The community’s concerns about pill burden, quality of care, financial barriers, TB etiology, stigma, and preference for pluralistic care need to be addressed to improve early detection. Health education messages should emphasize the curability of TB, the feasibility of treatment and the engagement of traditional healers as partners in identifying cases and facilitating adherence to treatment.
Knowledge and awareness of tuberculosis among Roma population in Belgrade: a qualitative study	Vukovic D.S. [50]	2011	Tuberculosis (TB) remains an important health problem in the Roma population in Serbia. Recent studies have highlighted the importance of increasing awareness of TB and reducing the associated stigmas to reduce the incidence of TB, and enable earlier diagnosis and effective treatment. This study investigated the knowledge and beliefs about transmission, symptoms, and treatment of TB, as well as attitudes towards patients with TB among the Roma population in Belgrade. The focus-group method was considered appropriate for investigating knowledge and beliefs about TB. A total of 24 Roma people aged 19–55 years participated in three focus-group discussions. All participants knew that TB was a pulmonary disease and could be contagious. Saliva was the most commonly mentioned mode of transmission. Some individuals thought, albeit hesitantly, that TB could be transmitted by shaking hands with an infected individual. Of factors contributing to TB, participants mentioned bad living conditions, low quality and lack of food, and stress. Participants quoted chest pain, cough, hemoptysis, loss of appetite, loss of weight, weakness, and sweating as basic symptoms of TB. Participants believed that effective treatment should include resting, taking prescribed medicines, inhaling fresh air, and eating “strong” food, such as bacon and pork; these approaches were considered as important as taking antibiotics. In addition, participants mentioned that they use some folk medicines. Relatives and friends, and to a lesser extent, television, were the main sources of information about TB. Participants most appreciate personal contact with doctors as a source of information. They concluded that participants were aware of the seriousness TB as well as some of the modes of transmission; however, they had some misconceptions. An important finding was the confidence in doctors expressed by the Roma people.
Quality of life of patients with scabies	Jin-gang A. [51]	2010	Scabies is a highly contagious disease caused by the mite *Sarcoptes scabiei*, and the disease is still a major public health problem in many resource-poor regions. Apart from the skin lesions or substantial morbidity, scabies also leads to social stigma. However, quality of life (QoL) has not been investigated in patients with scabies. The aim of this study was to assess the impact of scabies on patients’ QoL using the Dermatology Life Quality Index (DLQI) questionnaire and assess its feasibility and internal consistency. One hundred consecutive outpatients seeking treatment for scabies in the Department of Dermatology, the Second Hospital of Xi’an Jiaotong University, were assessed for eligibility for this prospective study from 8 August, 2008 to 20 December, 2008. Sulfur (10%) was selected in the treatment of scabies. A total of 96 patients completed the study. Among them, 78 (81.25%) of patients were considered cured at the end of the study. The mean ± SD DLQI score in our study was 10.09 ± 5.96. QoL of most of (71.9%) our patients have moderately affected. Questions 1 (symptoms), 2 (embarrassment), 7 (work or study), and 9 (sexual difficulties) had the most impact on patients with scabies. Domain 1 (symptoms and feelings) and 5 (personal relationships) scored higher than other domains. The Mann–Whitney U-test was used to test the equality of distributions of quantitative outcomes. The mean DLQI score before and after treatment was analyzed using Student’s *t*-test. The relationships between DLQI scores and clinical, social, and demographic factors were analyzed using ordinal multiple logistic regression. Construct validity was tested by factor analysis. Reliability was assessed by average inter-item correlation, item total correlation and Cronbach’s alpha.10 All analyses were performed using SPSS software (version 13.0; SPSS Inc., Chicago, IL, USA). *p* < 0.05 was interpreted as statistically significant. There was significant progress of QoL after treatment in their patients. No strong relationship between disease-related characteristics and QoL could be found. Scabies moderately affected the QoL of the patients. Sulfur could be considered as an effective treatment for patients with scabies.
Measuring stigma associated with tuberculosis and HIV⁄AIDS in southern Thailand: exploratory and confirmatory factor analyses of two new scales	Van Rie A. [52]	2008	The aim of this study is to develop scales to measure tuberculosis and HIV⁄AIDS stigma in a developing world context. A cross-sectional study of tuberculosis patients in southern Thailand was done, where participants were asked to rate their agreement with items measuring TB and HIV⁄AIDS stigma. Developing the scales involved exploratory and confirmatory factor analyses, internal consistency, construct validity, test–retest reliability, and standardized summary scores. Factor analyses identified two sub-scales associated with both tuberculosis and HIV⁄AIDS stigma: community and patient perspectives. Goodness-of-fit was good (Tucker & Lewis Index—TLI = 94, Comparative Fit Index—CFI = 0.88 and Root-Mean-Square Error of Approximation—RMSEA = 0.11), internal consistency was excellent (Cronbach’s alphas 0.82–0.91), test–retest reliability was moderate, and construct validity showed an inverse correlation with social support. These scales have good psychometric properties that measure stigma associated with tuberculosis and HIV⁄AIDS and allow assessment of stigma from community and patient perspectives. Their use will help document the burden of stigma, guide the development of interventions, and evaluate stigma reduction programs in areas with a high HIV⁄AIDS and tuberculosis burden.
Stigma Scale Revised: Reliability and Validity of a Brief Measure of Stigma for HIV Youth	Wright K. [27]	2007	The purpose of this study was to shorten a human immunodeficiency virus (HIV) stigma scale to make it less burdensome for HIV-positive (HIV) youth without compromising psychometric properties. Youth infected with HIV were participants in a clinical trial investigating the efficacy of a motivational intervention to improve condom use and prevent or decrease substance use. Youth were recruited from an adolescent HIV clinic within a tertiary care children’s hospital located in a major metropolitan area. Inclusion criteria included HIV status, ages 16–25 years, and English speaking. The sample for the larger study consisted of 64 participants. They added the stigma questionnaire later in the study and had 48 clients complete this measure. The sample was 88% African American; 52% male, 46% female, and 2% male to female transgender; 64% of the males self-identified as gay or bisexual. The majority (86%) of the youth were infected through sexual contact. In our study population, 50% scored above the clinical cut-off for the General Severity Index, 42% for depression, and 42% for anxiety. The shortened questionnaire showed good internal consistency and validity, suggesting that a 10-item measure of stigma has promise for assessing this important construct in HIV youth.
The experience of SARS-related stigma at Amoy Gardens	Lee S. [29]	2005	Severe Acute Respiratory Syndrome (SARS) possesses characteristics that render it particularly prone to stigmatization. SARS-related stigma, despite its salience for public health and stigma research, has had little examination. This study combines survey and case study methods to examine subjective stigma among residents of Amoy Gardens (AG), the first officially recognized site of community outbreak of SARS in Hong Kong. A total of 903 residents of AG completed a self-report questionnaire derived from two focus groups conducted toward the end of the three-month outbreak. Case studies of two residents who lived in Block E, the heart of the SARS epidemic at AG, complement the survey data. In total, 41.0% and 59.0% of respondents were male and female, respectively. They were aged 15–80 years (mean 39.1 years with most between 35 and 54). Most were employed (86.9%). In total, 47 respondents (5.2%) were reportedly ex-SARS patients. Moreover, 128 respondents (15.2%) reported having had SARS symptoms only. A total of 68 respondents (7.7%) reported the presence of confirmed SARS cases in their household that resulted in 10 deaths. Of the four housing clusters, those from Cluster 1 (including 60 Block E residents) gave the highest response rate (38.0% cf. 20.1%, 28.5%, 13.3% for the other clusters). There was no significant difference in the sociodemographic characteristics of respondents from different clusters. However, as expected, more from Cluster 1 were ex-SARS patients (12.3% vs. <1.2% among the others, χ^2^ = 55.33, degrees of freedom—df = 3, *p* < 0.001) or had ex-SARS household members (17.5% vs. <2.3% among the others, χ^2^ = 73.90, df 1⁄4 3, *p* < 0.001). Findings show that stigma affected most residents and took various forms of being shunned, insulted, marginalized, and rejected in the domains of work, interpersonal relationships, use of services and schooling. Stigma was also associated with psychosomatic distress. Resident strategies for diminishing stigma varied with gender, age, education, occupation, and proximity to perceived risk factors for SARS, such as residential location, previous SARS infection, and the presence of ex-SARS household members. Residents attributed stigma to government mismanagement, contagiousness of the mysterious SARS virus, and alarmist media reporting. Stigma clearly decreased, but never completely disappeared, after the outbreak. The findings confirm and add to existing knowledge on the varied origins, correlates, and impacts of stigma. They also highlight the synergistic roles of inconsistent health policy responses and risk miscommunication by the media in rapidly amplifying stigma toward an unfamiliar illness. While recognizing the intrinsically stigmatizing nature of public health measures to control SARS, we recommend that a consistent inter-sectoral approach is needed to minimize stigma and to make an effective health response to future outbreaks.
